# Use of the Arabin pessary in women at high risk for preterm birth: long-term experience at a single tertiary center in Malaysia

**DOI:** 10.1186/s12884-021-03838-x

**Published:** 2021-05-10

**Authors:** Rahana Abd Rahman, Ixora Kamisan Atan, Anizah Ali, Aida Mohd Kalok, Nor Azlin Mohamed Ismail, Zaleha Abdullah Mahdy, Shuhaila Ahmad

**Affiliations:** Department of Obstetrics & Gynaecology, Faculty of Medicine, Universiti Kebangsaan Malaysia Medical Center, Jalan Yaacob Latif, Bandar Tun Razak, Cheras, 56000 Kuala Lumpur, Malaysia

**Keywords:** Arabin pessary, High risk, Preterm birth, Progestogen

## Abstract

**Background:**

Spontaneous preterm birth is a global issue that contributed to perinatal morbidities and mortalities worldwide. The study aimed to describe the experience at UKM Medical Center in managing women at high risk for spontaneous preterm birth using the Arabin pessary.

**Methods:**

This is a retrospective observational study involving 58 pregnancies from 1st January 2013 to 31st December 2019. Inclusion criteria were previous mid-trimester miscarriage and/or preterm birth, previous cervical surgery or short cervical length on routine sonogram. The demographic data, characteristics of each pregnancy and details of outcomes and management were described.

**Results:**

The majority of women were Malay with mean age and body mass index of 32.9 ± 4.2 years and 27.1 ± 6.3 kg/m^2^ respectively. The most frequent indications for Arabin pessary insertion were previous mid-trimester miscarriage (46.4%) and early preterm birth (17.2%). A total of 73.4% of these women had the pessary inserted electively at a mean cervical length of 31.6 ± 9.1 mm at median gestation of 15.0 weeks. They were managed as outpatient (56.9%), inpatient (24.1%) or mixed (19.0%) with combination of progestogen (81.0%) and 53.4% received antenatal corticosteroids. Spontaneous preterm birth at or more than 34 weeks gestation occurred in 74.1% with birthweight at or more than 2000 g (82.4%). Despite cervical funneling in 12 women (20.7%), 66.7% delivered at or later than 34 weeks gestation and 2 (16.7%) resulted in miscarriage.

**Conclusions:**

Insertion of the Arabin pessary is beneficial to prevent spontaneous preterm birth in pregnant women who are at high risk. In particular, early insertion and close monitoring allows the best possible outcomes.

**Trial registration:**

This study was retrospectively registered with ClinicalTrials.gov (NCT04638023) on 20/11/2020.

## Background

Preterm birth is defined by delivery before 37 completed weeks and is a major cause of perinatal morbidity and mortality [1]. Consequences of preterm birth are among the most common cause of death in children under 5 years of age [2]. The global incidence of preterm birth in 2010 was 11.1% with the highest rate in low income countries [2]. Worldwide, the rate remained the same in most countries but seemed to be increased in the United States [3, 4]. Likewise, the rate of preterm birth in Malaysia increased between 2010 (8.1%) and 2012 (11.3%) [5]. The cost of management of preterm infants is massive with the mean cost highest in infants with birthweight of less than 1000 g [5].

Preterm birth can either be iatrogenic due to severe preeclampsia and fetal growth restriction or spontaneous caused by vaginal infection, and cervical incompetence [6–9]. Spontaneous preterm labor contributed to 40–45% of preterm birth [10]. The diagnosis of cervical incompetence is difficult and suspected either from history or incidental finding of ultrasound measurement of short cervical length with or without funnelling. It is characterized by painless dilatation of the cervix as the pregnancy advances. Typically, the gestation of birth reduces with increasing numbers of pregnancy and the duration of labor is short. The pathophysiology is poorly understood and hence the difficulty to predict and treat the condition effectively. To date, the strongest predictor is short cervical length on transvaginal sonogram before 28 weeks of gestation and the risk increased with decreasing length and presence of funnelling [11].

Cervical insufficiency is a condition believed to be caused by congenital deficiency of collagen in the cervical tissue or from cervical trauma. Consequently, the cervix is unable to withstand the pressure from the growing fetus in utero causing the cervix to shorten and open creating a funnel. Traditionally cervical cerclage is recommended in this group of women based on either history alone and/or cervical assessment in the present pregnancy [12]. However, this method is operator dependent, requires hospital admission, operating theatre for local or general anesthesia, and causes cervical scarring. In addition, the data on the efficacy in preventing preterm birth is contradicting [13]. There had been robust research to investigate an alternative treatment using a vaginal pessary. Arabin pessary use in women at high risk of preterm birth had been shown to have promising outcomes [14, 15]. Despite the conflicting results in the earlier years, studies that are more recent had shown positive outcomes [16–19]. On the other hand, a systematic review by Conde-Agudelo A et al. did not support the use of cervical pessary in singleton or twin pregnancies amongst high risk women to prevent spontaneous preterm birth [20]. The mechanism of action of cervical pessary is to shift the cervix posteriorly to deviate the uterine load to the anterior lower segment and at the same time pressuring it to remain closed [21].

The aim of this study was to describe the experience of managing women who were at high risk for spontaneous preterm birth using Arabin pessary.

## Methods

### Setting and eligibility criteria

This was a single tertiary center retrospective analysis which included 66 pregnancies that underwent Arabin pessary insertion (Dr Arabin, Witten, Germany) between 1st January 2013 until 31st December 2019 in UKM Medical Center. Ethics approval had been obtained through the Institutional Ethics and Research Board (FF-2017-372). No consent was required by the ethics committee as this was a retrospective study. Administrative permissions and/or licenses were not required by our team to access the data used in our research.

This study had been performed in accordance with the ethical standards as laid down in the 1964 Declaration of Helsinki. The women included in this study had singleton pregnancies who were high risk for spontaneous preterm birth based on previous spontaneous preterm birth before 34^+ 0^ weeks or previous cervical surgery such as large loop excision of transformation zone (LLETZ) or loop electrosurgical excision procedure (LEEP) with their gestational age confirmed via dating scans in the first trimester. Patients were excluded if the details of the pregnancy were incomplete, had Arabin pessary inserted in other centers or delivered elsewhere.

#### Management protocol

Patients who were identified to be high risk for spontaneous preterm birth based on the obstetrics history were identified. Screening for vaginal infections was performed by taking vaginal swab samples which were sent for microscopic, cultural and sensitivity analysis (HVS C&S). Women with candidosis were treated with antifungal pessary according to our routine protocol. If the diagnosis of cervical insufficiency was highly suggestive, the Arabin pessary was inserted electively at 14 to 16 weeks gestation regardless of the cervical length measurement. If the diagnosis of cervical incompetence was doubtful, the patients’ cervical length was monitored via transvaginal sonogram initiated from 14 weeks. It was performed every 2 weeks until 28 to 30 weeks gestation. The pessary was inserted if there was cervical length shortening irrespective of whether there were any symptoms. The decision to insert the pessary and subsequent management such as additional progestogen therapy, in- or out-patient management and antenatal corticosteroids were based on the attending obstetricians. The progestogen therapy was administered either parenterally (250 – 500 mg weekly) or vaginally (200 mg daily before bedtime) based on the physician’s choice. Transvaginal sonogram to measure the cervical length was performed using a 7-MHz transducer (model D8-4U-Resona 7; Mindray, Shen Zhen, China) according to the Fetal Medicine Foundation (FMF) guideline. All the patients were followed up 2 to 4 weekly after pessary insertion in the antenatal clinic until delivery. Progestogen therapy was stopped at 34 weeks gestation and Arabin pessary was removed at 36 weeks or more or when the patients were in established labor.

All of the records were traced and scrutinized including the sociodemographic variables which consist of maternal age, race, pre-pregnancy body mass index (BMI), parity, number of miscarriages, indication for Arabin pessary insertion, and intervention for previous preterm birth. The clinical characteristics included cervical length and gestational age at insertion, presence of funnelling, whether pessary was inserted electively or as emergency, continuity of management post insertion as inpatient, outpatient or mixed, addition of progestogen therapy, presence of antenatal complications such as diabetes, hypertension, anemia, threatened preterm labor and preterm prelabor rupture of membranes, administration of corticosteroids, pathological smear and gestational age at removal. Primary outcome measured was birth at or more than 34 weeks gestation. Secondary outcome measured were type of labor, mode of delivery, neonatal birthweight and neonatal intensive care unit (NICU) admission.

Miscarriage was defined as pregnancy loss at 22 weeks and less [22]. Preterm birth was defined as birth before 37 completed week [1]. Early neonatal death was defined as death between 0 to 7 days of life [23].

### Statistical analysis

Data were analyzed using SPSS software package version 23 (IBM SPSS Statistics, Armonk, NY, USA). Simple descriptive analyses were used to describe the demographic data and clinical characteristics of the study group. Comparisons between groups were made with Fisher’s exact test or contingency coefficient for dichotomous data and Student’s t-test and non-parametric test for normally and not normally distributed data respectively. *p* value of < 0.05 was deemed significant.

## Results

A total of 66 pregnancies were identified whereof eight were excluded as they were either transferred to another center for delivery due to financial constraint or the data was incomplete. The remaining data of 58 pregnancies were analyzed.

Table 1 demonstrates the demographic data of study population. The majority of patients was multiparous (56/58, 96.6%) Malay (42/58, 72.4%) women with mean maternal age and body mass index (BMI) of 32.9 ± 4.2 years and 27.1 ± 6.3 kg/m^2^ respectively. The most common indication for insertion of an Arabin pessary was previous mid-trimester miscarriages (27/58, 46.4%) and there were 15 patients (15/58, 25.9%) who had been managed with either Arabin pessary or cervical cerclage in previous pregnancies. There was a total of 12 patients (12/58, 20.7%) who had been managed with cervical cerclage in their previous pregnancies.

**Table 1:** Demographic data of study population at UKM Medical Center from 1st January 2013 until 31st December 2019, *n* = 58

Mean maternal age ± SD, (years)	32.9 ± 4.2
Race, n (%)	
Malay	42 (72.4)
Chinese	10 (17.2)
Indian	1 (1.7)
Others	5 (8.6)
Mean pre-pregnancy BMI ± SD, (kg/m^2^)	27.1 ± 6.3
Parity, n (%)	
Nulliparous	2 (3.4)
Multiparous	56 (96.6)
Number of miscarriages, n (%)	
0–2	46 (79.3)
≥ 3	12 (20.7)
Indications, n (%)	
Previous mid-trimester miscarriage (A)	27 (46.4)
Previous early preterm birth (B)	10 (17.2)
Short cervical length on ultrasound	8 (13.8)
Previous cervical surgery or trauma	3 (5.2)
A & B	10 (17.2)
Previous intervention, n (%)	
Arabin pessary	6 (10.3)
Cervical cerclage	12 (20.7)
Progestogen only	2 (3.4)

*SD* standard deviation, *BMI* body mass index

Table 2 demonstrates that most patients (42/58, 72.4%) had elective Arabin pessary insertion at the median gestation of 15.0 (13.75–20.3) weeks with mean cervical length of 31.6 ± 9.1 mm. Slightly more than half (33/58, 56.9%) were managed as outpatient throughout their pregnancies and (31/58, 53.4%) received antenatal corticosteroids. The majority of them (47/58, 81.0%) had a combination of progestogen therapy either parenteral or vaginally and 51.7% (30/58) of patients had associated pathological smear. Among the most common findings were presence of group B *Streptococcus* (24/58, 41.4%) followed by *Candida albicans* (6/58, 10.2%).

**Table 2:** Clinical characteristics of study population, at UKM Medical Center from 1st January 2013 until 31st December 2019, *n* = 58

Mean cervical length at insertion ± SD, (mm)	31.6 ± 9.1
Presence of funneling, n (%)	12 (20.7)
Median gestation at insertion, weeks (IQR)	15.0 (13.8–20.3)
Type of insertion, n (%)	
Elective	42 (72.4)
Emergency	16 (27.6)
Subsequent management, n (%)	
Outpatient	33 (56.9)
Inpatient	14 (24.1)
Mixed	11 (19.0)
Combination with progestogen, n (%)	
Proluton	36 (61.1)
Uterogestan	11 (19.0)
Complications, n (%)	
Diabetes	14 (24.1)
Hypertension	1 (1.7)
Anaemia	3 (5.2)
Threatened preterm labor	2 (3.4)
Preterm prelabor rupture of membranes	2 (3.4)
Antenatal corticosteroids, n (%)	31 (53.4)
Pathological vaginal smear, n (%)	
Group B streptococcus	24 (41.4)
*Candida albicans*	6 (10.2)
Median gestational age of Arabin pessary removal, weeks (IQR)	35.9 (29.0–36.0)

*IQR* inter quartile range

Table 3 shows 29/58 (50.0%) pregnancies delivered more than 37 weeks gestation and 22/58 (37.9%) had preterm livebirths. The total of preterm birth at or more than 34 weeks gestation was 74.1% (43/58). Amongst the preterm livebirths, the majority was late (14/22, 63.6%) followed by extreme (5/22, 22.7%), moderate (2/22, 9.1%) and very (1/22, 4.6%) preterm birth. In accordance with this, most neonates were born with a birthweight of 2000 g or more (42/51, 82.4%) followed by less than 1000 g (4/51, 7.8%). There was one stillbirth delivered at 24^+ 4^ weeks with birthweight of 620 g following an emergency insertion of pessary at 21 weeks gestation for short cervical length on ultrasound. Majority of the patients had spontaneous labor (48/52, 92.3%) and 34/52 (65.4%) delivered vaginally. Emergency caesarean section was performed in 13/18 (72.2%) of pregnancies due to fetal distress, malpresentation and refusal for vaginal birth after a caesarean section.

**Table 3:** Pregnancy outcome in study population at UKM Medical Center from 1st January 2013 until 31st December 2019

Birth outcome, *n* = 58, n (%)	
> 37 weeks gestation	29 (50.0)
Preterm livebirth (24^+ 0^ to 36^+ 6^)	22 (37.9)
Preterm stillbirth	1 (1.8)
Miscarriage	6 (10.3)
Total birth ≥34 weeks gestation	43 (74.1)
Type of labor, *n* = 52, n (%)	
Spontaneous	48 (92.3)
Induction of labor	4 (7.7)
Mode of delivery, *n* = 52, n (%)	
Spontaneous vaginal	34 (65.4)
Instrumental	0 (0)
Caesarean section	18 (34.6)
Elective	5 (27.8)
Emergency	13 (72.2)
Median gestational age at birth, weeks (IQR)	37.0 (33.6–37.7)
Preterm live birth (weeks), n (%)	22
Late (34–36^+ 6^)	14 (63.6)
Moderate (32–33^+ 6^)	2 (9.1)
Very (28–31^+ 6^)	1 (4.6)
Extreme (< 28)	5 (22.7)
Median birthweight, (g) (IQR)	2700.0 (1842.0–2920.0)
Live birthweight, (g), n (%)	51
≥ 2000	42 (82.4)
1500–1990	3 (5.8)
1000–1490	2 (4.0)
< 1000	4 (7.8)
NICU admission, n (%)	11 (19.0)

*NICU* neonatal intensive care unit

Table 4 demonstrates the clinical characteristics and outcomes of patients with cervical funnelling. Majority of them had emergency insertion of the pessary with shorter mean cervical length (19.9 ± 6.4 mm) at median gestation of 21.0 weeks (IQR 18.0–24.3). Not surprisingly, most of these patients were managed as inpatient (6/12, 50.0%) or mixed in- and outpatient (4/12, 33.3%). Almost all of them were administered with progestogen therapy and interestingly, 75.0% (9/12) were complicated with diabetes.

**Table 4:** Clinical characteristics and pregnancy outcomes of patients with funneling at UKM Medical Center from 1st January 2013 until 31st December 2019, *n* = 12

Insertion type, n (%)	
Elective	3 (25.0)
Emergency	9 (75.0)
Mean cervical length at insertion ± SD, (mm)	19.9 ± 6.4
Median gestation at insertion, weeks (IQR)	21.0 (18.0–24.3)
Subsequent management, n (%)	
Outpatient	2 (16.7)
Inpatient	6 (50.0)
Mixed	4 (33.3)
Combination with progestogen, n (%)	
Proluton	9 (75.0)
Uterogestan	2 (16.7)
Complications, n (%)	
Diabetes	9 (75.0)
Thyroid disorders	2 (16.7)
Preterm prelabor rupture of membranes	1 (8.3)
Birth outcome, n (%)	
> 37 weeks gestation	7 (58.3)
Preterm livebirth (24^+ 0^ to 36^+ 6^)	3 (25.0)
Miscarriage	2 (16.7)
Total birth ≥34 weeks gestation	8 (66.7)
Median birthweight, (g) (IQR)	2775.0 (1285.0–2962.5)
Live birthweight, (g), n (%)	
≥ 2000	7 (70.0)
1500–1990	1 (10.0)
1000–1490	2 (20.0)
NICU admission, n (%)	3 (30.0)

Table 5 compares pregnancies that had elective versus emergency insertion of the Arabin pessary. There was significant difference in mean cervical length at insertion (34.7 ± 7.6 mm vs 23.5 ± 7.9 mm, *p* < 0.001), median gestation at insertion (14.0 weeks vs 21.5 weeks, *p* < 0.001) and presence of funneling (7.1% vs 56.2%, *p* < 0.001) between those who had elective and emergency pessary insertion respectively. There were no significant differences in mean maternal age, mean pre-pregnancy BMI, pregnancy outcome, birth less than 34 weeks gestation, median gestational age at birth, median birthweight and rate of NICU admission.

**Table 5:** Comparison between elective and emergency insertion of Arabin pessary in study population at UKM Medical Center from 1st January 2013 until 31st December 2019, n = 58

	Elective insertion ***n*** = 42	Emergency insertion ***n*** = 16	OR (95% CI) ***p*** value
Mean maternal age ± SD (years)	32.6 ± 4.4	33.7 ± 3.7	0.4^a^
Mean pre-pregnancy body mass index ± SD, kg/m^2^	27.8 ± 6.4	25.0 ± 5.5	0.2^a^
Presence of funnelling, n (%)			16.7 (3.6–77.5)
No	39 (92.9)	7 (43.8)	< 0.001^b^
Yes	3 (7.1)	9 (56.2)	
Mean cervical length at insertion ± SD, (mm)	34.7 ± 7.6	23.5 ± 7.9	< 0.001^a^
Median gestation at insertion, weeks (IQR)	14.0 (13.0–15.0)	21.5 (18.8–25.0)	< 0.001^c^
Pregnancy outcome, n (%)			0.2^d^
Term livebirth	22 (52.4)	7 (43.8)	
Preterm livebirth	15 (35.7)	7 (43.8)	
Miscarriage	5 (11.9)	1 (6.3)	
Early neonatal death	0 (0.0)	1 (6.1)	
Birth < 34 weeks gestation, n (%)	10 (23.8)	5 (31.3)	1.4 (0.4–5.2) 0.7^b^
Median gestational age at birth, weeks (IQR)	37.0 (34.0–38.5)	36.5 (28.4–37.3)	0.8^c^
Median birthweight, (g) (IQR)	2630.0 (1897.5–2925.0)	2700.0 (1152.5–2937.5)	0.8^c^
NICU admission, n (%)	8 (21.6)	3 (23.1)	1.1 (0.2–4.9) 1.0^b^

^a^Student’s t-test, ^b^Chi square test, difference expressed in odds ratio (95% CI), ^c^Non-parametric test, ^d^contingency coefficient, df = 2

## Discussion

This is the first study that was conducted in Malaysia that showed the experience of a single tertiary center in using Arabin pessary in women at high risk for spontaneous preterm birth. In our center, the pessary has been used as an alternative to cervical cerclage since 2013. Patients with obstetric history that was highly suggestive of cervical insufficiency had elective Arabin pessary insertion at 14 to 16 weeks. Emergency insertion was performed in those who had asymptomatic or symptomatic cervical length shortening on ultrasound.

There are only a few studies that recruited the same cohort of patients similar to ours. The rate of preterm birth less than 34 and 37 weeks in our cohort was 25.9 and 50% respectively. Hence, our median gestation at insertion was earlier (15.0 weeks) with longer mean cervical length (31.6 mm) than the studies done by Ivandic et al. and Barinov et al. [14, 24]. Likewise, the majority of neonates delivered in this study weighed 2000 g or more. This was in accordance with a study by Ivandic et al. whereby the rate was 29 and 50% respectively [14]. Majority had Arabin pessary insertion at 20 weeks gestation but the cervical length was not mentioned [14]. Barinov et al. recruited women at high risk for spontaneous preterm birth and randomized them into Arabin pessary and progestogen or circular cervical cerclage and progestogen at 11 to 22 weeks gestation [24]. They demonstrated no significant differences in the rate of preterm birth. The preterm birth rate was higher as compared to our study at 44.4% before 34 weeks and concentrated on the neonatal outcomes instead of the details in the management [24]. These studies differed to ours as most of our patients had the pessary inserted electively before the onset of cervical shortening.

The data in this study had demonstrated that majority of preterm births were classified under late with birthweight of 2000 g or more. The results of this study cannot be compared to other published data as the cohort of patients recruited was different. In our center, patients who were identified as high risk for spontaneous preterm labor would undergo cervical length monitoring from as early as 14 weeks gestation. Those with history that was suggestive of cervical incompetence would have the Arabin pessary insertion electively at 14 weeks. On the other hand, those whose histories were not clear, were counselled and options were given for them to choose. Either the Arabin pessary would be inserted electively at 14 weeks or inserted only in the presence of cervical length shortening following 2 weekly cervical length measurements from 14 to 28 weeks gestation.

The protocol of the management of women at high risk for spontaneous preterm birth in our center is unique. Most studies recruited low risk women with shortened cervical length following screening of asymptomatic low risk women via transvaginal sonogram. The results were contradicting. Goya *et.al* and Saccone *et.al* both recruited this cohort of women at 18 to 22 weeks gestation. They demonstrated significantly lower rate of preterm birth and higher birthweight as compared to the control group [25, 26]. Hui *et.al* and Nicolaides *et.al* recruited similar cohort of women at 20 to 24 weeks and randomized them into control and treated groups. Both studies did not show reduction of spontaneous preterm birth less than 34 weeks as compared to the control group [27, 28].

We are aware that our study was limited by the small sample size and this was not a randomized control trial. We did not include data on the neonatal outcomes in regard to incidence of perinatal morbidities such as necrotizing enterocolitis, intraventricular hemorrhage, respiratory distress syndrome, retinopathy of prematurity, and duration of NICU stay. Additionally, the added use of progestogen might have influenced the outcomes of this study. However, the selected population were highly selective consisting of those who were at high risk of preterm birth which is one of the reasons there was no control group in this study. As this was a single center experience, which targeted a homogenous group of women, the management did not differ much between the obstetricians involved.

## Conclusions

The results of our observational study suggested that the insertion of the Arabin pessary in women at high risk for spontaneous preterm birth is beneficial if it is inserted electively earlier at 14 weeks gestation. We found that the early elective insertion and close monitoring of the patients are beneficial in the management of this high risk group of women to allow additional steps in management aiming for the best outcome. Arabin pessary is safe with minimal side effects, affordable, simple to use and non-operator dependent. A larger multi-center study is required for further validation of our results.

## Data Availability

The datasets used and/or analyzed during the current study are available from the corresponding author on reasonable request.

## Abbreviations

UKM: Universiti Kebangsaan Malaysia
LLETZ: Large Loop Excision of Transformation Zone
LEEP: Loop Electrosurgical Excision Procedure
HVS C&S: High Vaginal Swab Culture & Sensitivity
FMF: Fetal Medicine Foundation
BMI: Body Mass Index
NICU: Neonatal Care Intensive Care Unit
